# Diphenyl (isopropyl­amido)­phosphate

**DOI:** 10.1107/S1600536812047940

**Published:** 2012-11-28

**Authors:** Fahimeh Sabbaghi, Mehrdad Pourayoubi, Marek Nečas, Michal Babiak

**Affiliations:** a Department of Chemistry, Zanjan Branch, Islamic Azad University, Zanjan, Iran; b Department of Chemistry, Ferdowsi University of Mashhad, Mashhad, Iran; c Department of Chemistry, Faculty of Science, Masaryk University, Kotlarska 2, Brno CZ-61137, Czech Republic

## Abstract

The P atom in the title compound, C_15_H_18_NO_3_P, is in a distorted tetra­hedral P(O)(O)_2_N environment; the bond angles at P are in the range 98.16 (6)–115.82 (6)°. In the crystal, adjacent mol­ecules are linked *via* N—H⋯O=P hydrogen bonds into a chain running parallel to the *b* axis. The methyl groups are disordered over two sets of sites in a 0.677 (14):0.323 (14) ratio. The crystal studied was a non-merohedral twin with a refined minor component of 22.31 (4)%.

## Related literature

For bond lengths and angles in a related structure, see: Sabbaghi *et al.* (2011 ▶).
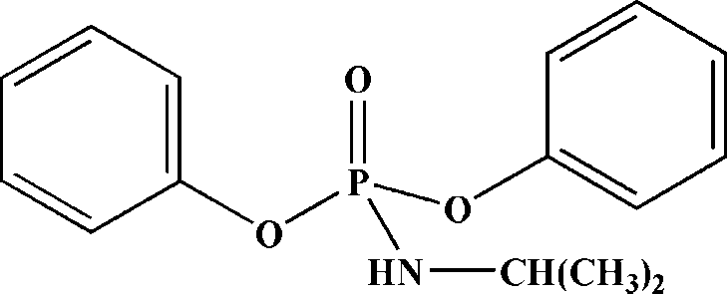


## Experimental

### 

#### Crystal data

C_15_H_18_NO_3_P
*M* *_r_* = 291.27
Monoclinic, 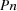

*a* = 8.4432 (5) Å
*b* = 5.3030 (4) Å
*c* = 16.3443 (11) Å
β = 90.453 (6)°
*V* = 731.78 (9) Å^3^
*Z* = 2
Mo *K*α radiation
μ = 0.19 mm^−1^
*T* = 120 K
0.45 × 0.42 × 0.40 mm

#### Data collection

Oxford Diffraction Xcalibur (Sapphire2) diffractometer
Absorption correction: multi-scan (*CrysAlis RED*; Oxford Diffraction, 2009 ▶) *T*_min_ = 0.918, *T*_max_ = 0.926
6226 measured reflections
6226 independent reflections
6040 reflections with *I* > 2σ(*I*)

#### Refinement

*R*[*F* ^2^ > 2σ(*F* ^2^)] = 0.027
*wR*(*F* ^2^) = 0.081
*S* = 1.06
6226 reflections
208 parameters
28 restraints
H atoms treated by a mixture of independent and constrained refinement
Δρ_max_ = 0.17 e Å^−3^
Δρ_min_ = −0.18 e Å^−3^
Absolute structure: Flack (1983 ▶), 1229 Friedel pairs
Flack parameter: 0.05 (6)

### 

Data collection: *CrysAlis CCD* (Oxford Diffraction, 2009 ▶); cell refinement: *CrysAlis CCD*; data reduction: *CrysAlis RED* (Oxford Diffraction, 2009 ▶); program(s) used to solve structure: *SHELXS97* (Sheldrick, 2008 ▶); program(s) used to refine structure: *SHELXL97* (Sheldrick, 2008 ▶); molecular graphics: *Mercury* (Macrae *et al.*, 2008 ▶); software used to prepare material for publication: *SHELXTL* (Sheldrick, 2008 ▶) and *enCIFer* (Allen *et al.*, 2004 ▶).

## Supplementary Material

Crystal structure: contains datablock(s) I, global. DOI: 10.1107/S1600536812047940/ff2090sup1.cif

Structure factors: contains datablock(s) I. DOI: 10.1107/S1600536812047940/ff2090Isup2.hkl

Additional supplementary materials:  crystallographic information; 3D view; checkCIF report

## Figures and Tables

**Table 1:** Hydrogen-bond geometry (Å, °)

*D*—H⋯*A*	*D*—H	H⋯*A*	*D*⋯*A*	*D*—H⋯*A*
N1—H1*N*⋯O3^i^	0.81 (1)	2.23 (1)	3.0065 (17)	161 (2)

Symmetry code: (i) 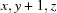.

**Fig. 1.**
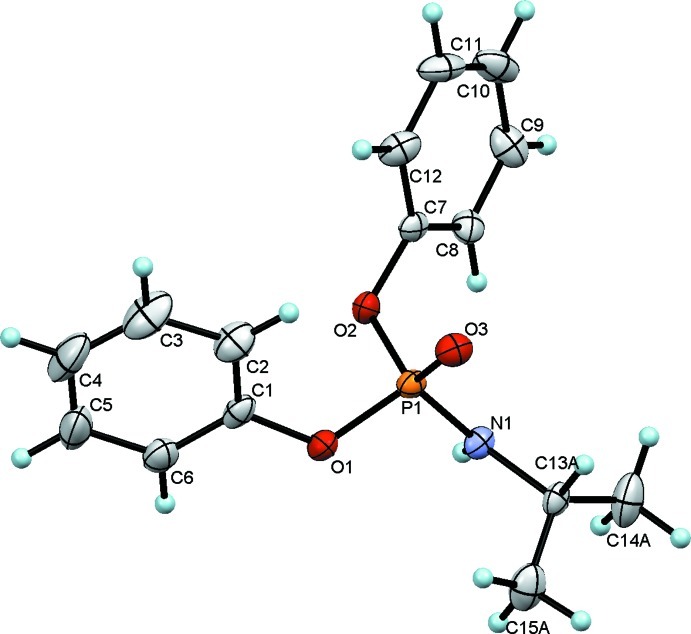
The molecular structure of the title compound with ellipsoids shown at the 50% probability level and H atoms are drawn as small spheres of arbitrary radii. The minor component of disordered part has been omitted for clarity and only one orientation is shown for the disordered part.

## supplementary crystallographic information

### Comment

This work is a continuation of our studies of phosphoramidate compounds, during which the structures of various diphenyl amido phosphates, for example [C_6_H_5_O]_2_P(O)[NHCH(C_2_H_5_)(C_6_H_5_)] (Sabbaghi *et al.*, 2011) were reported. Here, we report the synthesis and crystal structure determination of the title compound, [C_6_H_5_O]_2_P(O)[NHCH(CH_3_)_2_].

The P═O (1.4602 (11) Å), P—O (1.5858 (11) and 1.5896 (11) Å) and P—N (1.6043 (14) Å) bond lengths are within the expected values (Sabbaghi *et al.*, 2011).

The P atom adopts a distorted tetrahedral configuration (Fig. 1). The bond angles at the P atom vary in the range 98.16 (6) [O1—P1—O2] to 115.82 (6)° [O3—P1—O2].

The C—O—P bond angles (124.07 (10) [C1—O1—P1] and 121.74 (10)° [C7—O2—P1]) and the C13A—N1—P1 (124.19 (11)°) bond angle are standard for this category of phosphoramidate compounds (Sabbaghi *et al.*, 2011).

In the crystal structure, molecules are linked *via* N—H···O═P hydrogen bonds into extended chains running parallel to the *b* axis (Table 1).

### Experimental

To a solution of [C_6_H_5_O]_2_P(O)Cl (2 mmol) in dry CH_3_CN (30 ml), a solution of isopropylamine (4 mmol) in the same solvent (5 ml) was added at ice bath temperature under stirring. After 4 h, the solvent was removed and the product was washed with distilled water and recrystallized from CH_3_CN/n-C_6_H_14_ (4:1) at room temperature. The single crystals suitable for X-ray analysis were obtained from this solution after a few days at room temperature.

### Refinement

The crystal sample was non-merohedrally twinned. Using data reduction software, a HKLF 5 file was produced for a two-component twin and used in the refinement. The fractional contribution of the minor twin component converged to 0.2231 (4). All carbon bound H atoms were placed at calculated positions and were refined as riding with their *U*_iso_ set to either 1.2*U*_eq_ or 1.5*U*_eq_ (methyl) of the respective carrier atoms; in addition, the methyl H atoms were allowed to rotate about the C—C bond. Nitrogen bound H atom was located in a difference Fourier map and its position was refined while the N—H distance was fixed at 0.88 Å and the *U*_iso_ set to 1.2*U*_eq_ of N1. The disordered methyl groups were modeled over two sites while restraining their anisotropic displacement parameters to be approximately isotropic (ISOR). To maintain a correct hydrogen geometry, a dummy atom with zero occupancy was created and constrained to share the same site (EXYZ) and anisotropic displacement parameters (EADP) with a fully occupied carbon atom bound to N1.

### Crystal data

**Table:**

C_15_H_18_NO_3_P	*F*(000) = 308
*M**_r_* = 291.27	*D*_x_ = 1.322 Mg m^−^^3^
Monoclinic, *P**n*	Mo *K*α radiation, λ = 0.71073 Å
*a* = 8.4432 (5) Å	Cell parameters from 3821 reflections
*b* = 5.3030 (4) Å	θ = 3.4–27.5°
*c* = 16.3443 (11) Å	µ = 0.19 mm^−^^1^
β = 90.453 (6)°	*T* = 120 K
*V* = 731.78 (9) Å^3^	Block, colourless
*Z* = 2	0.45 × 0.42 × 0.40 mm

### Data collection

**Table:**

Oxford Diffraction Xcalibur (Sapphire2) diffractometer	6226 independent reflections
Radiation source: Enhance (Mo) X-ray Source	6040 reflections with *I* > 2σ(*I*)
Graphite monochromator	*R*_int_ = 0.000
Detector resolution: 8.4353 pixels mm^-1^	θ_max_ = 25.0°, θ_min_ = 3.8°
ω scan	*h* = −10→10
Absorption correction: multi-scan (*CrysAlis RED*; Oxford Diffraction, 2009)	*k* = −6→6
*T*_min_ = 0.918, *T*_max_ = 0.926	*l* = −19→19
6226 measured reflections	

### Refinement

**Table:**

Refinement on *F*^2^	Secondary atom site location: difference Fourier map
Least-squares matrix: full	Hydrogen site location: inferred from neighbouring sites
*R*[*F*^2^ > 2σ(*F*^2^)] = 0.027	H atoms treated by a mixture of independent and constrained refinement
*wR*(*F*^2^) = 0.081	*w* = 1/[σ^2^(*F*_o_^2^) + (0.0626*P*)^2^ + 0.0183*P*] where *P* = (*F*_o_^2^ + 2*F*_c_^2^)/3
*S* = 1.06	(Δ/σ)_max_ < 0.001
6226 reflections	Δρ_max_ = 0.17 e Å^−^^3^
208 parameters	Δρ_min_ = −0.18 e Å^−^^3^
28 restraints	Absolute structure: Flack (1983), 1229 Friedel pairs
Primary atom site location: structure-invariant direct methods	Flack parameter: 0.05 (6)

### Special details

**Table:**

Geometry. All e.s.d.'s (except the e.s.d. in the dihedral angle between two l.s. planes) are estimated using the full covariance matrix. The cell e.s.d.'s are taken into account individually in the estimation of e.s.d.'s in distances, angles and torsion angles; correlations between e.s.d.'s in cell parameters are only used when they are defined by crystal symmetry. An approximate (isotropic) treatment of cell e.s.d.'s is used for estimating e.s.d.'s involving l.s. planes.
Refinement. Refinement of *F*^2^ against ALL reflections. The weighted *R*-factor *wR* and goodness of fit *S* are based on *F*^2^, conventional *R*-factors *R* are based on *F*, with *F* set to zero for negative *F*^2^. The threshold expression of *F*^2^ > σ(*F*^2^) is used only for calculating *R*-factors(gt) *etc*. and is not relevant to the choice of reflections for refinement. *R*-factors based on *F*^2^ are statistically about twice as large as those based on *F*, and *R*- factors based on ALL data will be even larger.

### Fractional atomic coordinates and isotropic or equivalent isotropic displacement parameters (Å^2^)

**Table:**

	*x*	*y*	*z*	*U*_iso_*/*U*_eq_	Occ. (<1)
P1	0.49359 (4)	0.20465 (6)	0.51025 (3)	0.01893 (9)	
O1	0.46890 (12)	0.20756 (19)	0.41396 (6)	0.0229 (3)	
O2	0.32869 (12)	0.3259 (2)	0.53519 (6)	0.0213 (2)	
O3	0.52704 (12)	−0.04455 (19)	0.54422 (6)	0.0247 (3)	
N1	0.62616 (15)	0.4126 (2)	0.53057 (8)	0.0201 (3)	
H1N	0.603 (2)	0.559 (3)	0.5224 (10)	0.024*	
C1	0.33996 (19)	0.0959 (3)	0.37348 (10)	0.0241 (4)	
C2	0.2687 (2)	−0.1207 (3)	0.40086 (13)	0.0346 (4)	
H2	0.3036	−0.2017	0.4496	0.042*	
C3	0.1433 (2)	−0.2176 (3)	0.35464 (15)	0.0454 (6)	
H3	0.0915	−0.3663	0.3727	0.054*	
C4	0.0931 (2)	−0.1044 (4)	0.28402 (14)	0.0489 (6)	
H4	0.0066	−0.1725	0.2537	0.059*	
C5	0.1686 (2)	0.1079 (4)	0.25735 (13)	0.0440 (5)	
H5	0.1359	0.1848	0.2075	0.053*	
C6	0.2921 (2)	0.2121 (3)	0.30207 (11)	0.0307 (4)	
H6	0.3430	0.3614	0.2838	0.037*	
C7	0.27851 (18)	0.3331 (3)	0.61695 (10)	0.0197 (4)	
C8	0.31935 (19)	0.5376 (3)	0.66473 (9)	0.0244 (4)	
H8	0.3868	0.6656	0.6441	0.029*	
C9	0.2607 (2)	0.5528 (3)	0.74285 (10)	0.0319 (4)	
H9	0.2874	0.6928	0.7765	0.038*	
C10	0.1630 (2)	0.3656 (4)	0.77258 (11)	0.0376 (5)	
H10	0.1232	0.3763	0.8267	0.045*	
C11	0.1238 (2)	0.1643 (3)	0.72383 (12)	0.0360 (5)	
H11	0.0562	0.0361	0.7443	0.043*	
C12	0.1817 (2)	0.1460 (3)	0.64507 (12)	0.0306 (4)	
H12	0.1547	0.0065	0.6113	0.037*	
C13A	0.79551 (19)	0.3558 (3)	0.54259 (10)	0.0212 (4)	
H13A	0.8017	0.1911	0.5722	0.025*	0.323 (14)
C14A	0.8579 (13)	0.557 (2)	0.6011 (9)	0.042 (3)	0.323 (14)
H14A	0.8401	0.7242	0.5773	0.064*	0.323 (14)
H14B	0.9716	0.5312	0.6104	0.064*	0.323 (14)
H14C	0.8021	0.5444	0.6533	0.064*	0.323 (14)
C15A	0.8791 (19)	0.324 (3)	0.4671 (9)	0.037 (3)	0.323 (14)
H15A	0.8307	0.1869	0.4355	0.056*	0.323 (14)
H15B	0.9902	0.2833	0.4788	0.056*	0.323 (14)
H15C	0.8736	0.4808	0.4354	0.056*	0.323 (14)
C13B	0.79551 (19)	0.3558 (3)	0.54259 (10)	0.0212 (4)	0.00
H13B	0.8085	0.2301	0.5878	0.025*	0.677 (14)
C14B	0.8824 (4)	0.5997 (8)	0.5652 (4)	0.0306 (11)	0.677 (14)
H14D	0.8633	0.7267	0.5227	0.046*	0.677 (14)
H14E	0.9963	0.5662	0.5696	0.046*	0.677 (14)
H14F	0.8434	0.6622	0.6177	0.046*	0.677 (14)
C15B	0.8680 (8)	0.2476 (13)	0.4620 (4)	0.0298 (11)	0.677 (14)
H15D	0.8160	0.0877	0.4482	0.045*	0.677 (14)
H15E	0.9818	0.2188	0.4701	0.045*	0.677 (14)
H15F	0.8517	0.3683	0.4174	0.045*	0.677 (14)

### Atomic displacement parameters (Å^2^)

**Table:**

	*U*^11^	*U*^22^	*U*^33^	*U*^12^	*U*^13^	*U*^23^
P1	0.01606 (19)	0.01911 (19)	0.02163 (19)	0.00093 (19)	0.00011 (14)	0.0017 (2)
O1	0.0195 (6)	0.0253 (6)	0.0238 (6)	−0.0048 (5)	−0.0003 (5)	−0.0003 (5)
O2	0.0173 (6)	0.0247 (6)	0.0218 (6)	0.0019 (5)	−0.0019 (4)	0.0017 (5)
O3	0.0239 (6)	0.0191 (6)	0.0313 (6)	0.0014 (4)	0.0029 (5)	0.0024 (5)
N1	0.0184 (7)	0.0154 (7)	0.0265 (8)	0.0024 (5)	−0.0008 (5)	0.0027 (6)
C1	0.0155 (8)	0.0276 (9)	0.0291 (10)	−0.0002 (7)	0.0017 (7)	−0.0118 (7)
C2	0.0318 (11)	0.0258 (9)	0.0462 (12)	−0.0045 (8)	−0.0004 (8)	−0.0038 (9)
C3	0.0349 (12)	0.0286 (11)	0.0727 (17)	−0.0092 (9)	0.0004 (11)	−0.0148 (11)
C4	0.0245 (11)	0.0615 (14)	0.0604 (15)	−0.0062 (10)	−0.0054 (10)	−0.0292 (11)
C5	0.0239 (11)	0.0722 (16)	0.0359 (12)	0.0046 (10)	−0.0064 (8)	−0.0101 (11)
C6	0.0207 (10)	0.0402 (11)	0.0313 (10)	0.0021 (8)	0.0023 (8)	−0.0036 (9)
C7	0.0137 (9)	0.0204 (8)	0.0251 (9)	0.0058 (6)	0.0003 (7)	0.0059 (7)
C8	0.0262 (10)	0.0223 (8)	0.0248 (9)	−0.0025 (7)	−0.0027 (7)	0.0031 (7)
C9	0.0431 (12)	0.0242 (10)	0.0284 (10)	0.0084 (9)	−0.0014 (8)	0.0006 (8)
C10	0.0368 (12)	0.0450 (12)	0.0312 (10)	0.0186 (9)	0.0138 (9)	0.0091 (9)
C11	0.0289 (11)	0.0336 (11)	0.0457 (13)	0.0015 (8)	0.0162 (9)	0.0143 (9)
C12	0.0237 (10)	0.0255 (9)	0.0427 (11)	0.0001 (7)	0.0052 (8)	0.0003 (8)
C13A	0.0166 (9)	0.0217 (8)	0.0253 (9)	0.0034 (6)	−0.0049 (7)	0.0023 (7)
C14A	0.033 (4)	0.043 (4)	0.051 (4)	0.008 (3)	−0.019 (3)	−0.007 (3)
C15A	0.030 (4)	0.038 (5)	0.043 (4)	0.010 (4)	−0.006 (3)	−0.011 (4)
C13B	0.0166 (9)	0.0217 (8)	0.0253 (9)	0.0034 (6)	−0.0049 (7)	0.0023 (7)
C14B	0.0200 (15)	0.0309 (18)	0.041 (2)	0.0000 (12)	−0.0056 (15)	−0.0077 (16)
C15B	0.0230 (18)	0.035 (3)	0.0317 (19)	−0.0010 (19)	0.0069 (14)	−0.007 (2)

### Geometric parameters (Å, º)

**Table:**

P1—O3	1.4602 (11)	C9—C10	1.381 (3)
P1—O1	1.5858 (11)	C9—H9	0.9500
P1—O2	1.5896 (11)	C10—C11	1.371 (3)
P1—N1	1.6043 (14)	C10—H10	0.9500
O1—C1	1.4008 (19)	C11—C12	1.384 (2)
O2—C7	1.4057 (17)	C11—H11	0.9500
N1—C13A	1.4728 (19)	C12—H12	0.9500
N1—H1N	0.811 (13)	C13A—C15A	1.436 (15)
C1—C2	1.373 (2)	C13A—C14A	1.524 (8)
C1—C6	1.378 (2)	C13A—H13A	1.0000
C2—C3	1.393 (3)	C14A—H14A	0.9800
C2—H2	0.9500	C14A—H14B	0.9800
C3—C4	1.366 (3)	C14A—H14C	0.9800
C3—H3	0.9500	C15A—H15A	0.9800
C4—C5	1.367 (3)	C15A—H15B	0.9800
C4—H4	0.9500	C15A—H15C	0.9800
C5—C6	1.383 (3)	C14B—H14D	0.9800
C5—H5	0.9500	C14B—H14E	0.9800
C6—H6	0.9500	C14B—H14F	0.9800
C7—C12	1.367 (2)	C15B—H15D	0.9800
C7—C8	1.379 (2)	C15B—H15E	0.9800
C8—C9	1.376 (2)	C15B—H15F	0.9800
C8—H8	0.9500		
			
O3—P1—O1	114.19 (6)	C12—C7—O2	119.04 (14)
O3—P1—O2	115.82 (6)	C8—C7—O2	118.95 (13)
O1—P1—O2	98.16 (6)	C9—C8—C7	118.79 (15)
O3—P1—N1	114.26 (7)	C9—C8—H8	120.6
O1—P1—N1	106.55 (6)	C7—C8—H8	120.6
O2—P1—N1	106.26 (6)	C8—C9—C10	120.31 (17)
C1—O1—P1	124.07 (10)	C8—C9—H9	119.8
C7—O2—P1	121.74 (10)	C10—C9—H9	119.8
C13A—N1—P1	124.19 (11)	C11—C10—C9	119.82 (16)
C13A—N1—H1N	116.9 (13)	C11—C10—H10	120.1
P1—N1—H1N	117.1 (13)	C9—C10—H10	120.1
C2—C1—C6	121.57 (16)	C10—C11—C12	120.63 (15)
C2—C1—O1	122.74 (15)	C10—C11—H11	119.7
C6—C1—O1	115.65 (14)	C12—C11—H11	119.7
C1—C2—C3	117.70 (19)	C7—C12—C11	118.59 (17)
C1—C2—H2	121.2	C7—C12—H12	120.7
C3—C2—H2	121.2	C11—C12—H12	120.7
C4—C3—C2	121.69 (19)	C15A—C13A—N1	113.1 (7)
C4—C3—H3	119.2	C15A—C13A—C14A	116.8 (6)
C2—C3—H3	119.2	N1—C13A—C14A	105.7 (4)
C3—C4—C5	119.3 (2)	C15A—C13A—H13A	106.9
C3—C4—H4	120.4	N1—C13A—H13A	106.9
C5—C4—H4	120.4	C14A—C13A—H13A	106.9
C4—C5—C6	120.8 (2)	H14D—C14B—H14E	109.5
C4—C5—H5	119.6	H14D—C14B—H14F	109.5
C6—C5—H5	119.6	H14E—C14B—H14F	109.5
C1—C6—C5	118.93 (17)	H15D—C15B—H15E	109.5
C1—C6—H6	120.5	H15D—C15B—H15F	109.5
C5—C6—H6	120.5	H15E—C15B—H15F	109.5
C12—C7—C8	121.86 (15)		
			
O3—P1—O1—C1	67.82 (12)	C2—C1—C6—C5	0.3 (3)
O2—P1—O1—C1	−55.32 (12)	O1—C1—C6—C5	177.98 (15)
N1—P1—O1—C1	−165.06 (11)	C4—C5—C6—C1	1.1 (3)
O3—P1—O2—C7	48.50 (13)	P1—O2—C7—C12	−95.01 (17)
O1—P1—O2—C7	170.46 (11)	P1—O2—C7—C8	89.47 (14)
N1—P1—O2—C7	−79.57 (12)	C12—C7—C8—C9	0.0 (2)
O3—P1—N1—C13A	30.26 (15)	O2—C7—C8—C9	175.40 (15)
O1—P1—N1—C13A	−96.81 (13)	C7—C8—C9—C10	0.2 (3)
O2—P1—N1—C13A	159.24 (12)	C8—C9—C10—C11	−0.4 (3)
P1—O1—C1—C2	−33.7 (2)	C9—C10—C11—C12	0.3 (3)
P1—O1—C1—C6	148.68 (12)	C8—C7—C12—C11	−0.1 (3)
C6—C1—C2—C3	−1.2 (3)	O2—C7—C12—C11	−175.45 (15)
O1—C1—C2—C3	−178.68 (16)	C10—C11—C12—C7	−0.1 (3)
C1—C2—C3—C4	0.7 (3)	P1—N1—C13A—C15A	80.0 (6)
C2—C3—C4—C5	0.7 (3)	P1—N1—C13A—C14A	−150.9 (7)
C3—C4—C5—C6	−1.7 (3)		

### Hydrogen-bond geometry (Å, º)

**Table:**

*D*—H···*A*	*D*—H	H···*A*	*D*···*A*	*D*—H···*A*
N1—H1*N*···O3^i^	0.81 (1)	2.23 (1)	3.0065 (17)	161 (2)

Symmetry code: (i) *x*, *y*+1, *z*.

## References

[bb1] Allen, F. H., Johnson, O., Shields, G. P., Smith, B. R. & Towler, M. (2004). *J. Appl. Cryst.* **37**, 335–338.

[bb2] Flack, H. D. (1983). *Acta Cryst.* A**39**, 876–881.

[bb3] Macrae, C. F., Bruno, I. J., Chisholm, J. A., Edgington, P. R., McCabe, P., Pidcock, E., Rodriguez-Monge, L., Taylor, R., van de Streek, J. & Wood, P. A. (2008). *J. Appl. Cryst.* **41**, 466–470.

[bb4] Oxford Diffraction (2009). *CrysAlis CCD* and *CrysAlis RED* Oxford Diffraction Ltd, Yarnton, England.

[bb5] Sabbaghi, F., Pourayoubi, M., Negari, M. & Nečas, M. (2011). *Acta Cryst.* E**67**, o2512. 10.1107/S1600536811034507 PMC3200690 22064755

[bb6] Sheldrick, G. M. (2008). *Acta Cryst.* A**64**, 112–122. 10.1107/S0108767307043930 18156677

